# Unshrinking the baby lung to calm the VILI vortex

**DOI:** 10.1186/s13054-022-04105-x

**Published:** 2022-08-07

**Authors:** Gary Nieman, Michaela Kollisch-Singule, Harry Ramcharran, Joshua Satalin, Sarah Blair, Louis A. Gatto, Penny Andrews, Auyon Ghosh, David W. Kaczka, Donald Gaver, Jason Bates, Nader M. Habashi

**Affiliations:** 1 Department of Surgery, SUNY Upstate Medical Center, SUNY Upstate, 750 East Adams St., Syracuse, NY 13210 USA; 2 Department of Medicine, University of Maryland, Baltimore, MD USA; 3 Departments of Anesthesia, Biomedical Engineering, and Radiology, University of Iowa, Iowa City, IA USA; 4 Department of Biomedical Engineering, Tulane University, New Orleans, LA USA; 5 Department of Medicine, University of Vermont, Burlington, VT USA

**Keywords:** Acute respiratory distress syndrome (ARDS), Ventilator-induced lung injury (VILI), Protective mechanical ventilation

## Abstract

A hallmark of ARDS is progressive shrinking of the ‘baby lung,’ now referred to as the ventilator-induced lung injury (VILI) ‘vortex.’ Reducing the risk of the VILI vortex is the goal of current ventilation strategies; unfortunately, this goal has not been achieved nor has mortality been reduced. However, the temporal aspects of a mechanical breath have not been considered. A brief expiration prevents alveolar collapse, and an extended inspiration can recruit the atelectatic lung over hours. Time-controlled adaptive ventilation (TCAV) is a novel ventilator approach to achieve these goals, since it considers many of the temporal aspects of dynamic lung mechanics.

## Introduction

The acute respiratory distress syndrome (ARDS) remains a significant clinical problem with primary management being supportive use of mechanical ventilation (MV) [1]. However, inappropriate use of MV may result in ventilator-induced lung injury (VILI), which significantly increases ARDS mortality [2]. The use of MV for ARDS patients must balance its life-preserving attributes against its potential for harm. Strategies for optimizing this balance may vary substantially across patients, and even within the same patient over a given clinical course.

### The shrinking baby lung

The ARDS lung has been conceptualized as being composed of two distinct, gravitationally separated compartments: (1) dependent regions consisting atelectatic and/or edematous airspaces and (2) normally inflated tissue in less dependent regions comprising the so-called baby lung [3]. This conceptualization led to the hypothesis that ventilating patients with ARDS using a reduced tidal volume (*V*_T_) would protect the baby lung from volutrauma caused by overdistension, while simultaneously allowing the atelectatic compartment to rest and [ideally] recover [4]. It was also assumed that an appropriate level of positive end-expiratory pressure (PEEP), based on oxygenation, would avoid atelectrauma [4, 5]. This ARDSNet method was studied in a NIH clinical trial in 2000 [4] and showed a significant reduction in ARDS mortality using volume control [assist-control] mode with a *V*_T_ of 6 mL kg^−1^ compared to 12 mL kg^−1^ of ideal body weight. The use of 6 mL kg^−1^ soon became the standard of care for patients with ARDS.

**Fig. 1:**
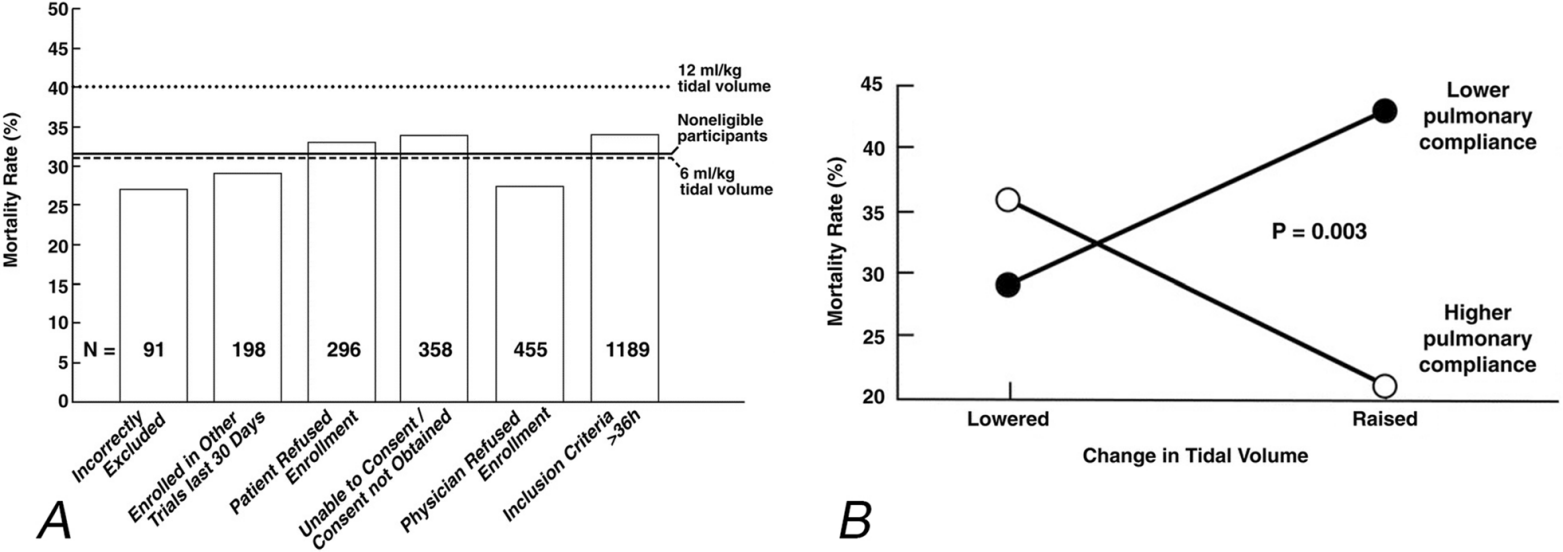
**A** Comparison of mortality rates in patients included and excluded from the ARDSNetwork low *V*_T_ trial (ARMA). The overall mortality rates of non-eligible patients who received standard of care mechanical ventilation (solid line; *n* = 2587), the 12 mL kg^−1^ tidal volume (*V*_T_) group (dotted line; *n* = 429), and the 6 mL kg^−1^ *V*_T_ group (dashed line; *n* = 432) are shown. Mortality was consistent across the non-eligible patients for the six exclusion reasons (vertical bars) and similar to that in the 6 mL kg^−1^ *V*_T_ group. Data provided to the Office of Human Research Protections from ARDSNet investigators from the ARMA trial for use at the June 9–11, 2003, consultants meeting. Available under the Freedom of Information Act [10]. **B** Pulmonary compliance plays a critical role in mortality with changes in *V*_T_ size. There was a significant interaction between pulmonary compliance and mortality rate in the ARMA trial (*p* = 0.003). Raising *V*_T_ increased mortality compared with lowering *V*_T_ (filled circles; 42% vs. 29%) in patients with lower pulmonary compliance. In contrast, raising *V*_T_ decreased mortality compared with lowering *V*_T_ (unfilled circles; 21% vs. 37%) in patients with higher pulmonary compliance [10]. (Permission to republish requested)

Recent statistical analyses suggest, however, that this low *V*_T_ (LV_T_) strategy has not lived up to its initial promise in reducing mortality in ARDS [6–9]. Deans et al. [10] analyzed data from 2587 patients that were screened but excluded from the ARDSNet Acute Respiratory Management Approach (ARMA) trial for technical reasons but were followed and treated with *V*_T_ of ~ 10 mL kg^−1^, which was the standard of care for ventilation at the time. The group with *V*_T_ ~ 10 mL kg had the same mortality as the LV_T_ (6 mL kg^1^) group (Fig. 1A). Also, a *V*_T_ greater than 6 mL kg^−1^ was not always associated with increased mortality, nor was LV_T_ always associated with reduced mortality. Rather, in patients with lower respiratory system compliance (*C*_RS_), raising *V*_T_ increased mortality compared to LV_T_ (42% for LV_T_ vs. 29% for high *V*_T_), while raising *V*_T_ in patients with higher *C*_RS_ reduced mortality (21% for high *V*_T_ vs. 37% for LV_T_; *p* = 0.003; Fig. 1B) [10].

These findings demonstrate that a patient’s individual lung pathophysiology is critically important for clinical outcome. Thus, there cannot be a single weight-based value of *V*_T_ that is best for all patients either at the initiation of MV or as their clinical course evolves. Appreciation of this fact is evidenced by the recent interest in driving pressure (Δ*P*), calculated as the difference between plateau and end-expiratory pressures. Δ*P* can evolve over a patient’s clinical course and is approximated by the ratio *V*_T_/*C*_RS_, which explains why letting *V*_T_ be influenced by *C*_RS_ has proven to be better at stratifying ARDS-related mortality risk compared to weight-based *V*_T_ [7, 11–15].

These observations beg the question as to whether Δ*P* should replace *V*_T_ as the key factor guiding protective ventilation strategies [7, 11–16]. Dramatic reductions in Δ*P* can be achieved with high-frequency oscillatory ventilation (HFOV), because it uses *V*_T_ that are less than the anatomic dead space volume. However, when tested in randomized controlled trials [17–19], HFOV failed to reduce ARDS-related mortality below that in the ARMA study [4]. Such a disappointing result may be related to the heterogeneous way that ventilation is distributed throughout the lung when cycled at high frequencies [20, 21] and the resultant heterogeneous distributions of parenchymal strain [20, 21]. Moreover, studies have shown that normal lung tissue, which is presumed to comprise the baby lung, is resistant to tissue damage induced by overdistension [22–30]. Also, although some studies suggest VILI occurs when a threshold of mechanical power applied to the lung is exceeded, studies in animal models suggest that VILI in the normal lung is only initiated when atelectrauma is allowed to occur regardless of *V*_T_ [26, 31, 32].

The baby lung concept, which established the rationale for LV_T_, was originally based on CT imaging [3]. However, more recent studies pairing CT with ^3^He or ^129^Xe magnetic resonance imaging (MRI) have shown that pathologic airspaces develop heterogeneously throughout the ARDS lung [33–36], contrary to the notion of a normal baby lung compartment. Furthermore, it has recently been shown that the principle mechanism of VILI at the tissue level is regional alveolar instability [5], defined as cyclic alveolar collapse causing scattered micro-atelectasis to develop throughout the lung [33–38]. In addition, Broche et al. using high-resolution synchrotron phase-contrast computerized tomography (CT) showed that acute lung injury significantly increased small airway (1.7–0.21 mm) closure and that this closure was time dependent. They suggest that the airway pressure release ventilation (APRV) mode, with a very short expiratory duration, may help to keep these small airways open [39]. Since micro-atelectasis cannot be seen in conventional chest radiographs or CT images [32, 40], its importance as a VILI mechanism may not have been fully appreciated until recently.

An assumption of the current protective ventilation approach is that the injured lung can be easily compartmentalized into simple opened and closed components. However, several studies have demonstrated that the injured lung has a far more heterogeneous distribution of parenchymal mechanical properties. Techniques that may be used to further assess such intraparenchymal heterogeneity include electrical impedance tomography [39, 41], computed tomographic image registration [42, 43] and oscillometric measurements of respiratory impedance [44]. How any of these approaches may be used to further refine the ventilation modality will of course require further investigation.

### Unshrinking the baby lung

**Fig. 2:**
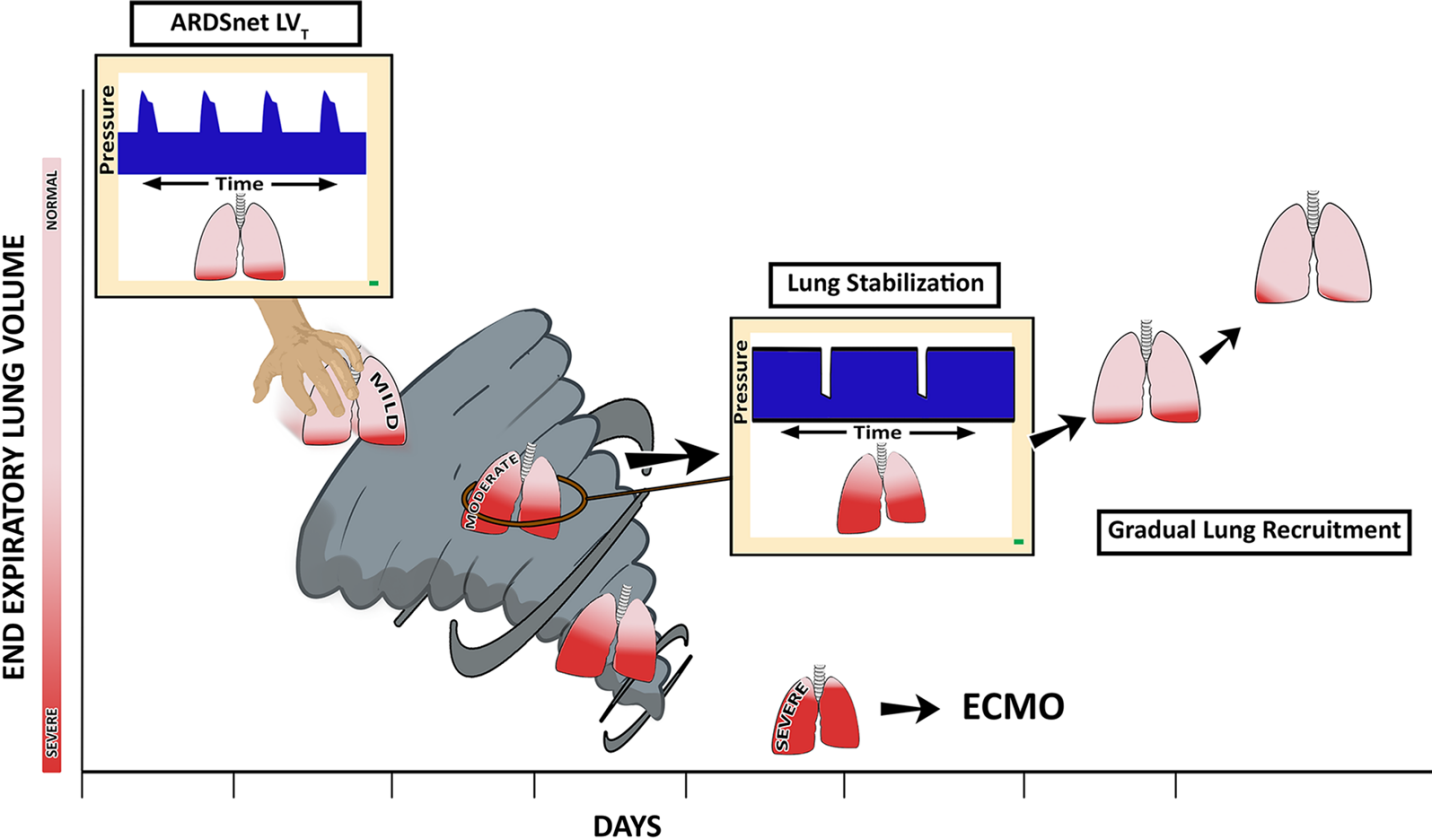
The evolution of ventilator-induced lung injury (VILI) can be described as an ever-shrinking baby lung known as a VILI vortex [45]. The ‘patient’ with mild ARDS with mostly open lung tissue (pink) and a lesser amount of collapsed tissue (red) is placed on ARDSNet LV_T_ ventilation. The LV_T_ strategy is designed to shield the ‘baby lung’ from overdistension. However, this strategy using low V_T_ and low airway pressures allows acutely injured tissue to continually collapse pushing it into the VILI vortex. As normal tissue progressively shrinks (pink → red), lung pathogenesis moves from mild-to-moderate ARDS. If unchecked, lung injury will progress into severe ARDS, at which point rescue methods such as extracorporeal membrane oxygenation (ECMO) may be necessary. ARDS causes the lung to become *time* and *pressure* dependent. This means that it will take more time for alveoli to open and less time for them to collapse at any given airway pressure. Thus, inspiratory and expiratory time can be used to accelerate alveolar opening and to minimize alveolar collapse. Using the ARDSNet approach, the short *time* at inspiration is not adequate to open collapsed alveoli, while the extended *time* at expiration will not prevent alveolar collapse (upper left ARDSNet LV_T_, Pressure/Time curve on the ventilator monitor). The open lung approach (OLA) using higher PEEP with and without recruitment maneuvers to rapidly (seconds or minutes) open the collapsed ARDS lung has not been successful at reducing ARDS-related mortality. Our group and others have shown the ability of inspiratory and expiratory duration to open and stabilize alveoli. Multiple studies using time-controlled ventilation strategies have confirmed that an extended inspiratory time will progressively recruit alveoli and a very brief expiratory time will prevent re-collapse [63, 66, 68, 71–83, 85, 86, 90]. An ventilator method to rapidly stabilize the lung (Center, Lung Stabilization, Pressure/Time curve on the ventilator monitor) using a very brief expiratory duration (Fig. 4B, Release Phase) has been shown to stabilize alveoli (Fig. 6, APRV 75%) and prevent progressive lung collapse pulling the lung from the Vortex. Once removed from the vortex, the collapsed tissue can be reopened slowly (gradual lung recruitment) over hours or day depending on the level of lung pathophysiology (Fig. 4B, CPAP Phase) [63, 66, 68, 71–83, 85, 86, 90]

Marini and Gattinoni recently described the progression of VILI as a *shrinking of the baby lung* whereby tissue moves from the open to the atelectatic compartment, a process labeled the ‘VILI vortex’ (Fig. 2) [45]. They hypothesize that as the baby lung continues to lose normal tissue as a result of alveolar instability and collapse, increasing stress and strain from a fixed *V*_T_ will be placed on the remaining open tissue, amplify existing lung injury. The VILI vortex concept was recently supported in a clinical study showing COVID-19-induced ARDS (CARDS) resulted in progressive lung collapse over a 3-week period [46].

**Fig. 3:**
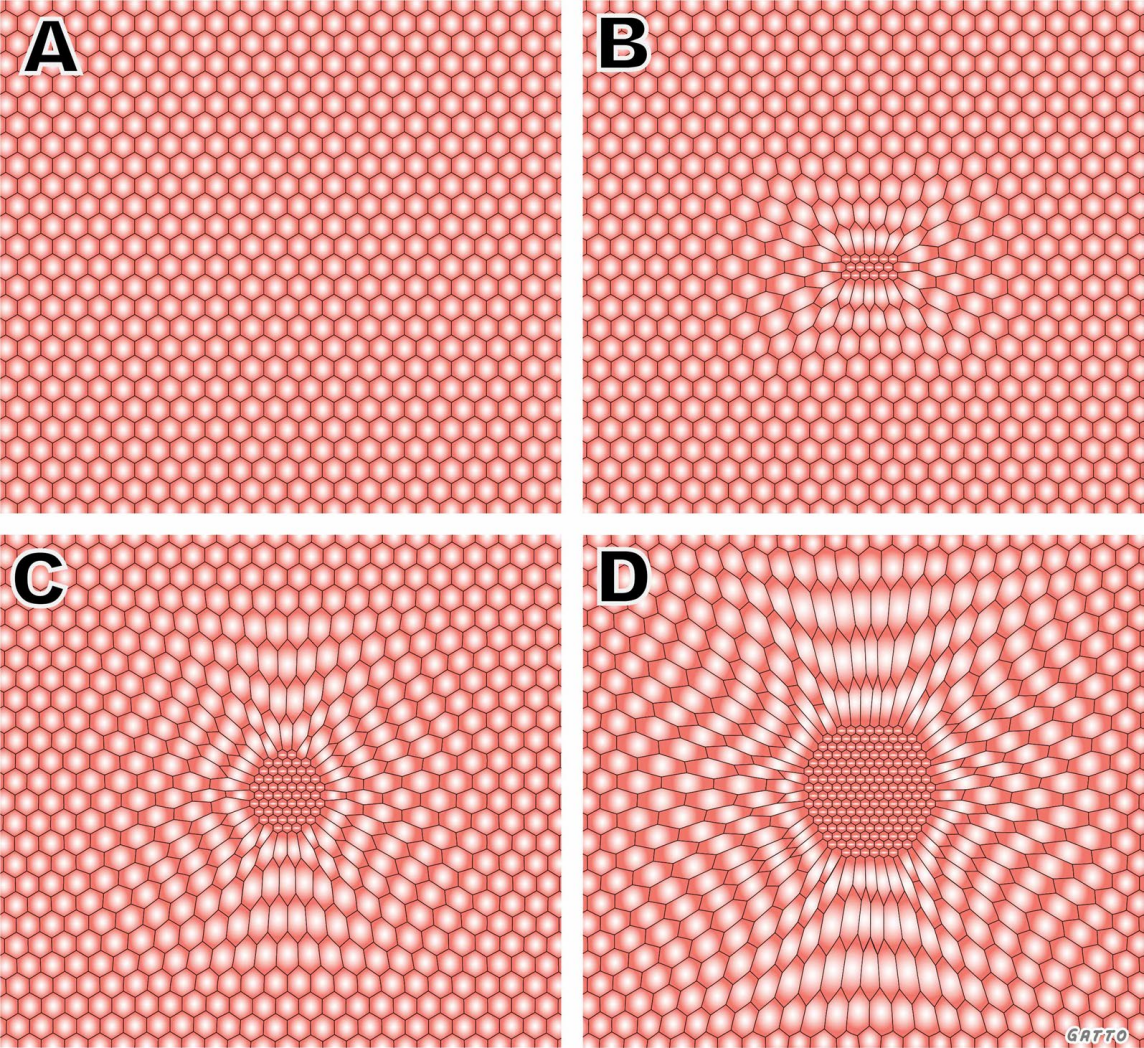
Ventilator-induced lung injury (VILI) in the microenvironment arises through a permeability-originated obstruction response (POOR) that is self-reinforcing (POOR-becomes-POORer). **A** In normal homogeneously ventilated lung, alveoli (hexagons) are uniformly open and stress is evenly distributed. **B** Isolated POOR areas of edema-filled or collapsed alveoli (center) that occur in early lung injury concentrate stress in adjacent patent alveoli, causing overdistension and instability. **C** The size of the POOR area expands due to collapse and flooding of surrounding alveoli, leading to the POOR region becoming POORer. **D** As the size of the POORer region expands, the stress applied to the surrounding alveoli is amplified and, unless this pathogenesis is interrupted, tissue damage secondary to VILI will continue to spread rapidly. (Permission to republish requested)

Unfortunately, fixing the VILI vortex is unlikely to be achieved solely with the use of a LV_T_ strategy because the parenchymal tissues at the interfaces between normal and atelectatic regions experience particularly high distortional forces. These forces predispose the interfacial tissues to being the site of VILI pathogenesis through a permeability-originated obstruction response (POOR) that is self-reinforcing (Fig. 3) [37]. This means that VILI will continue to progress at the interfacial regions even if the baby lung as a whole is not over-inflated. It also leads to the conjecture that the only way of preventing VILI progression is through a substantial reduction in the total burden of POOR regions, something that requires the immediate mitigation of alveolar instability followed by steps to progressively and safely reopen densely atelectatic lung tissue (Fig. 2) [38].

The above supposition is not new, and indeed, it motivated the open lung approach (OLA) to protective MV. The OLA attempts to open the majority of collapsed lung by applying high levels of PEEP, either with or without periodic recruitment maneuvers (RM) [47–50]. Several large clinical trials using the OLA, however, failed to show a reduction in ARDS-related mortality [47–50] despite preliminary data suggesting that opening the ARDS lung reduces VILI. While this might seem puzzling given that the OLA has an apparently well-founded physiologic rationale, there are two critical factors that potentially account for its lack of success. First, while recruitment/derecruitment (R/D) is a process normally associated with excursions in lung volume over the lower end of its functional range, these processes can manifest at increasingly elevated volumes as the lung becomes progressively more injured and may eventually even extend over the entire lung volume range [51, 52]. Thus, in some ARDS cases there may be no safe level of PEEP at which R/D is eliminated during MV.

The second factor not addressed by the OLA is that recruitment is not solely determined by the amount of airway pressure applied but also depends on inspiratory and expiratory time. Indeed, lung reopening may take a long time, sometimes hours or days, to occur [53]. The OLA uses RMs that rely on high inspiratory pressure applied transiently (seconds to minutes) to achieve rapid reopening, but lung recruitment can be challenging to achieve quickly even with very high pressures due to accumulation of surfactant deactivation and edema in the airspaces [38, 54]. Also, the nondurable recruitment produced by the OLA has the potential for increasing the number of unstable airspaces if adequate PEEP is not applied.

Similarly, reducing airway pressure may not cause immediate derecruitment because collapse also takes time to occur. The timescale for alveolar collapse is typically much faster than the timescale for alveolar reopening, and the delay before collapse begins may be < 0.5 s [55]. If PEEP is set too low and/or expiratory time too long, the resulting progressive lung collapse is reflected in transient increases in lung elastance (i.e., reductions in *C*_RS_) observed immediately following a RM [56]. Furthermore, the rates at which both alveolar opening and collapse occur are functions of the nature and severity of lung injury [57], as well as the level of airway pressure applied [58]. In general, the more severe the lung injury, the more rapid and extensive derecruitment will be at a given pressure [59]. As conventionally practiced, the administration of a RM does not consider how rapidly the lung derecruits following each maneuver. If atelectatic regions are recruited but not stabilized, then cyclic R/D will continue to cause atelectrauma [60].

The above considerations suggest that a non-injurious ventilation strategy for the ARDS lung must first be able to halt ongoing derecruitment and then progressively pull the lung out of the VILI vortex by gradually opening atelectatic lung (Fig. 2). To do this, such a strategy must have the following two key attributes:

- It must *rapidly stabilize alveoli* that are actively undergoing atelectrauma, such that susceptible airspaces do not have enough time to close during expiration and thus are prevented from having to reopen again during the subsequent inspiration. This prevents the accumulation of breath-to-breath atelectrauma and so pulls the lung from the VILI vortex.
- It must *progressively recruit atelectatic lung tissue* in a sustained manner over a period of hours or even days. This minimizes the amount of excessively distorted parenchyma at the interfaces between patent and atelectatic regions of the lung that are frequently the sites of VILI initiation [61]. Once gradual airspace opening begins, it can spread to adjacent collapsed regions via the forces of parenchymal tethering and interdependence [62].

**Fig. 4:**
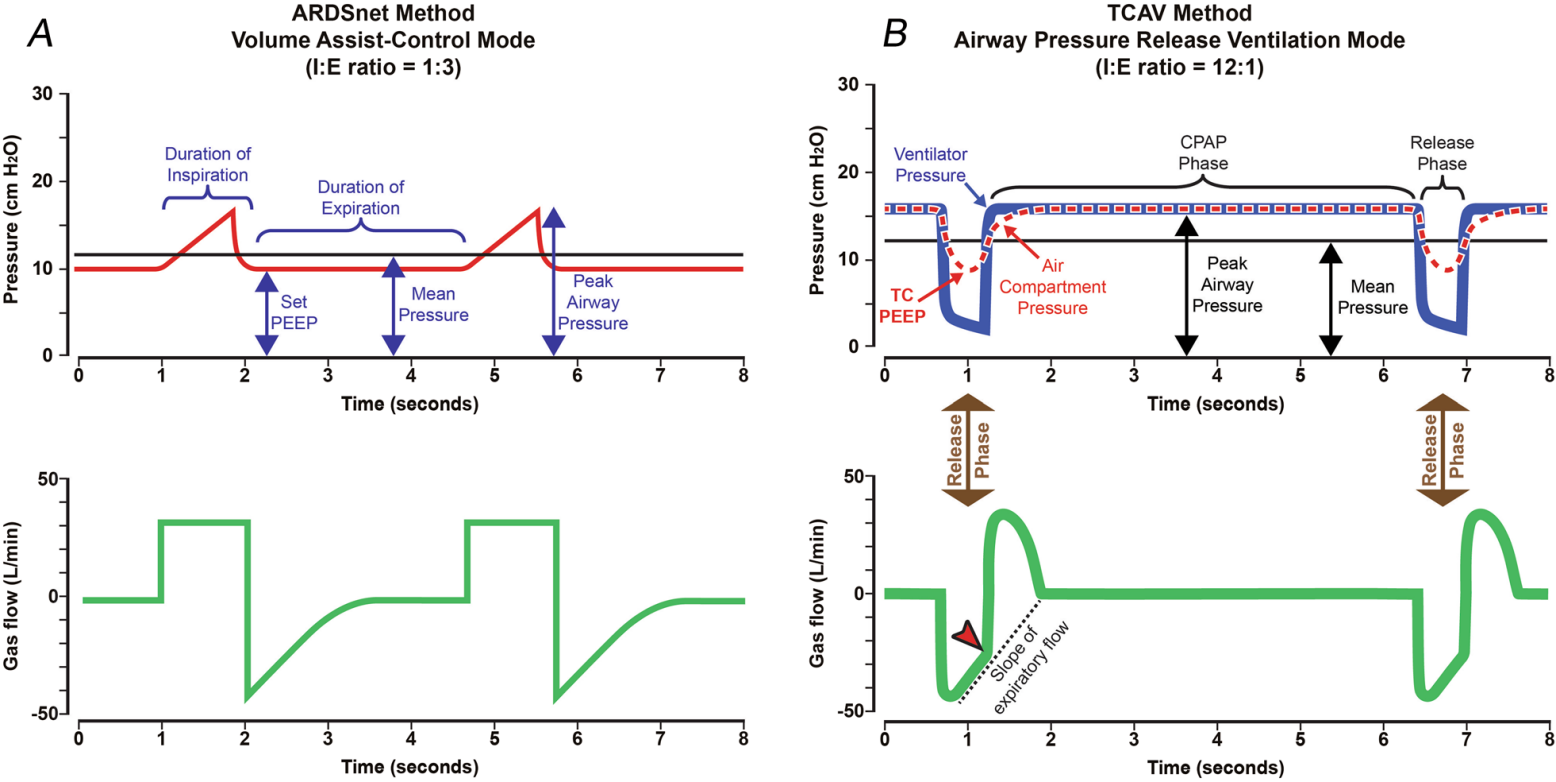
**A** Pressure/Time and Gas Flow/Time curves for Volume Assist-Control mode set and adjusted using the ARDSNet method. Key features include an inspiratory/expiratory ratio of 1:3. Plateau pressure is not extended, so peak inspiratory pressure is brief. Positive end-expiratory pressure (set-PEEP) and FiO_2_ are adjusted using oxygenation as the trigger for change [4]. **B** Pressure/Time and Gas Flow/Time curves for the airway pressure release ventilation (APRV) mode that is set and adjusted using the time-controlled adaptive ventilation (TCAV) method. Key features include an inspiratory/expiratory ratio as high as ~ 12:1, generating a prolonged inspiratory and short expiratory time. The continuous positive airway pressure (CPAP) phase is often ~ 90% of each breath. A tidal volume (*V*_T_) is not set, rather it is influenced by changes in (i) respiratory system compliance (*C*_RS_), (ii) the CPAP Phase pressure, and (iii) the duration of the Release Phase. The Release Phase is set as a percentage (75%) of the peak expiratory gas flow, which creates a very brief expiratory duration (Flow/Time curve, red arrowhead). Although this percentage is the same for most patients, the duration of the Release Phase can vary substantially in response to changes in *C*_RS_. The slope of the expiratory flow curve in the Gas Flow/Time curve provides a breath-to-breath measure of *C*_RS_. The lower the *C*_RS,_ the steeper the slope of the expiratory flow curve, and the shorter the Release Phase. The slope of the expiratory flow curve becomes less steep as the patient’s C_RS_ improves, which causes the Release Phase to lengthen (Fig. 7). The short Release Phase does not allow the lung time to depressurize fully, maintaining a time-controlled positive end-expiratory pressure (TC-PEEP, red dotted line). TC-PEEP is ~ 50% of the CPAP Phase pressure [91]. (Permission to republish requested)

**Fig. 5:**
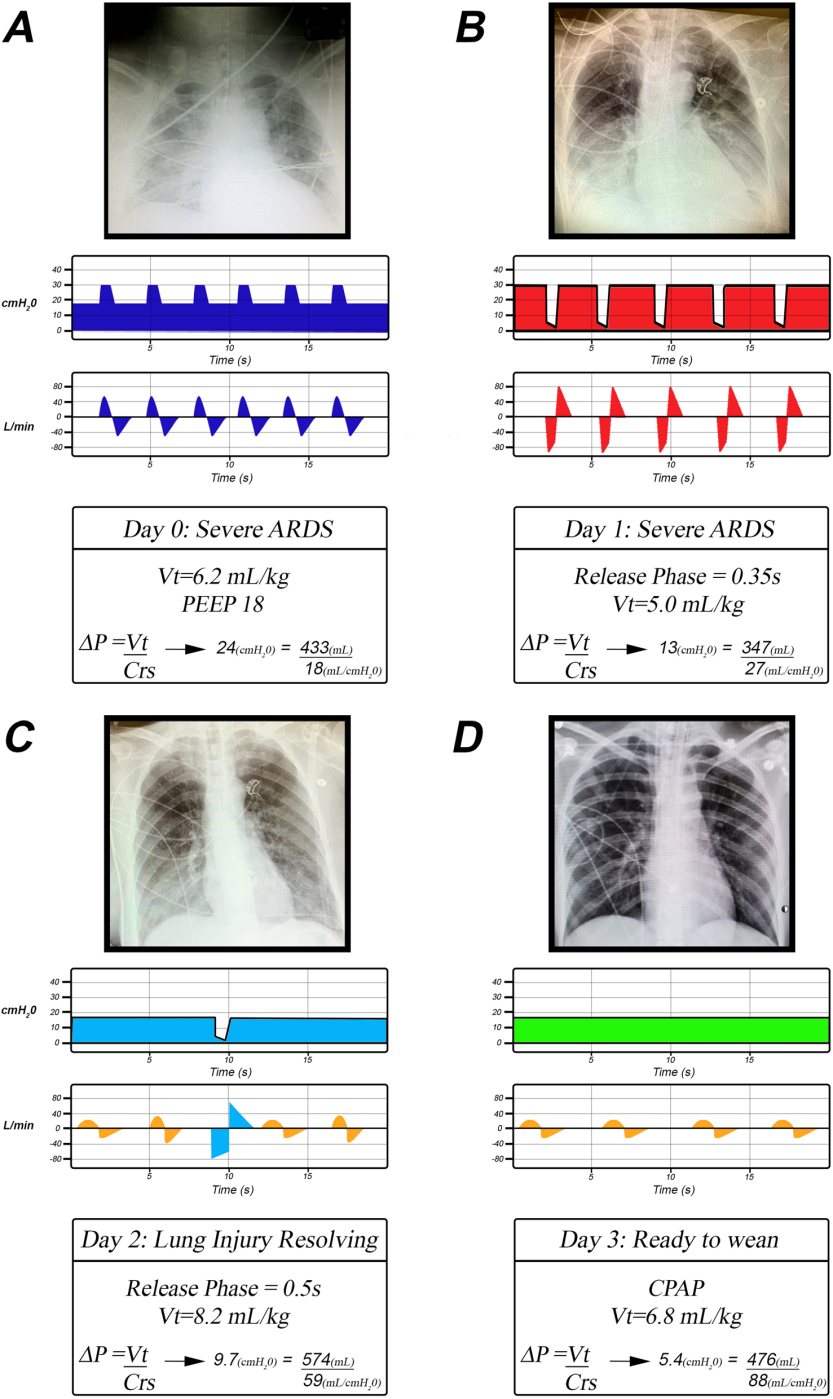
A representative illustration of the sequence of events typically seen during the progressive ‘Unshrinking the Baby Lung’ using the TCAV method to set and adjust the APRV mode. Using this method, the lung is first stabilized using a brief expiratory duration (**B**) and then gradually recruits collapsed lung tissue over hours or days using an extended inspiratory duration (**B**). **A** Patient with severe ARDS with extensive lung collapse and edema (X-ray) ventilated using the ARDSNet low *V*_T_ method showing typical Pressure/Time and Flow/Time curves (blue) as seen on a ventilator monitor. Even with a low *V*_T_ (6.2 mL kg^−1^), the driving pressure (Δ*P*) is elevated (24 cmH_2_O) because the *C*_RS_ is very low (18 mL/cmH_2_O). **B** The first day after the patient has been switched to the TCAV method, and the lung has begun to reopen showing typical Pressure/Time and Flow/Time curves as seen on a ventilator monitor (red). Although the lung has partially opened, it is still unstable and would rapidly re-collapse at lower airway pressures or if expiratory time was greater than the 0.35 s Release Phase. The *C*_RS_ has improved (27 mL cmH_2_O^−1^) because of the recruited lung tissue (X-ray). Nevertheless, the C_RS_ is low and lung recoil remains high, so the Release Phase is brief. The brief Release Phase generates a small *V*_T_ (5.0 mL kg^−1^), which maintains the Δ*P* (13 cmH_2_O) within the safe range. At this time, there are no spontaneous breathing efforts. **C** On Day 2, the lung is nearing full recruitment, as evidenced by a markedly improved chest X-ray. The patient is breathing spontaneously (Flow/Time curve gold waves) and is contributing to the total minute ventilation (MVe). Although the *T*_Low_ is still set to 75%, the release time is now 0.5 s and *V*_T_ have increased (8.2 mL kg^−1^) with a more fully recruited lung. The Δ*P* has decreased to 9.7 cmH_2_O, even with a *V*_T_ of 8.2 mL kg^−1^ because of an increase in *C*_RS_ (59 mL cmH_2_O^−1^). Only an occasional Release Phase is needed to facilitate CO_2_ removal (Flow/Time curve light blue wave), since most of the MVe is generated by the patient’s spontaneous breathing (Flow/Time curve gold waves). Spontaneous *V*_T_ average 6.0 mL kg. **D** On Day 3, the patient is ready to be weaned with restored lung volume. The Release Phase has been eliminated and the patient is generating all of their MVe with spontaneous breathing (Flow/Time curve gold waves). As a result, *V*_T_ has increased further (6.8 mL kg^−1^), and Δ*P* (5.4 cmH_2_O) and C_RS_ (88 mL cmH_2_O^−1^) are within their normal ranges. Note that a *V*_T_ greater than 6 mL kg^−1^ is not harmful (i.e., normal Δ*P*) when delivered into a fully inflated lung with high *C*_RS_ [10]. Also, note *V*_T_ remains proportional to *C*_RS_. *V*_T_ = tidal volume, PEEP = positive end-expiratory pressure, *C*_RS_ = respiratory system compliance, MVe = minute ventilation, and Δ*P* = Driving Pressure (*V*_T_/*C*_RS_).

Neither attribute is likely to be realized in the injured lung during conventional MV because expiration is usually long enough to allow rapidly closing lung units sufficient time to derecruit unless very high PEEP is applied, and inspiration is too brief to recruit collapsed alveoli that open slowly over time (Figs. 4A, 5A). The above two attributes can be realized, however, if the pattern of ventilation is allowed to depart from traditional mechanical ventilation in the following two ways (Figs. 4B, 5B):

1. Expiration must be sufficiently brief that derecruitment does not have enough time to occur prior to the beginning of the next inspiration (Fig. 4B, Release Phase).
2. Inspiratory duration and pressure must be sufficient to progressively recruit atelectatic lung over an extended period of time, but not so high as to be injurious to the parenchyma or have adverse hemodynamic consequences (Fig. 4B, CPAP Phase). Pressure must be sustained in a manner that is capable of recruiting lung units gradually, and this pressure must be applied for hours or days until the lung is fully open and stable (Fig. 5B–D).

**Fig. 6:**
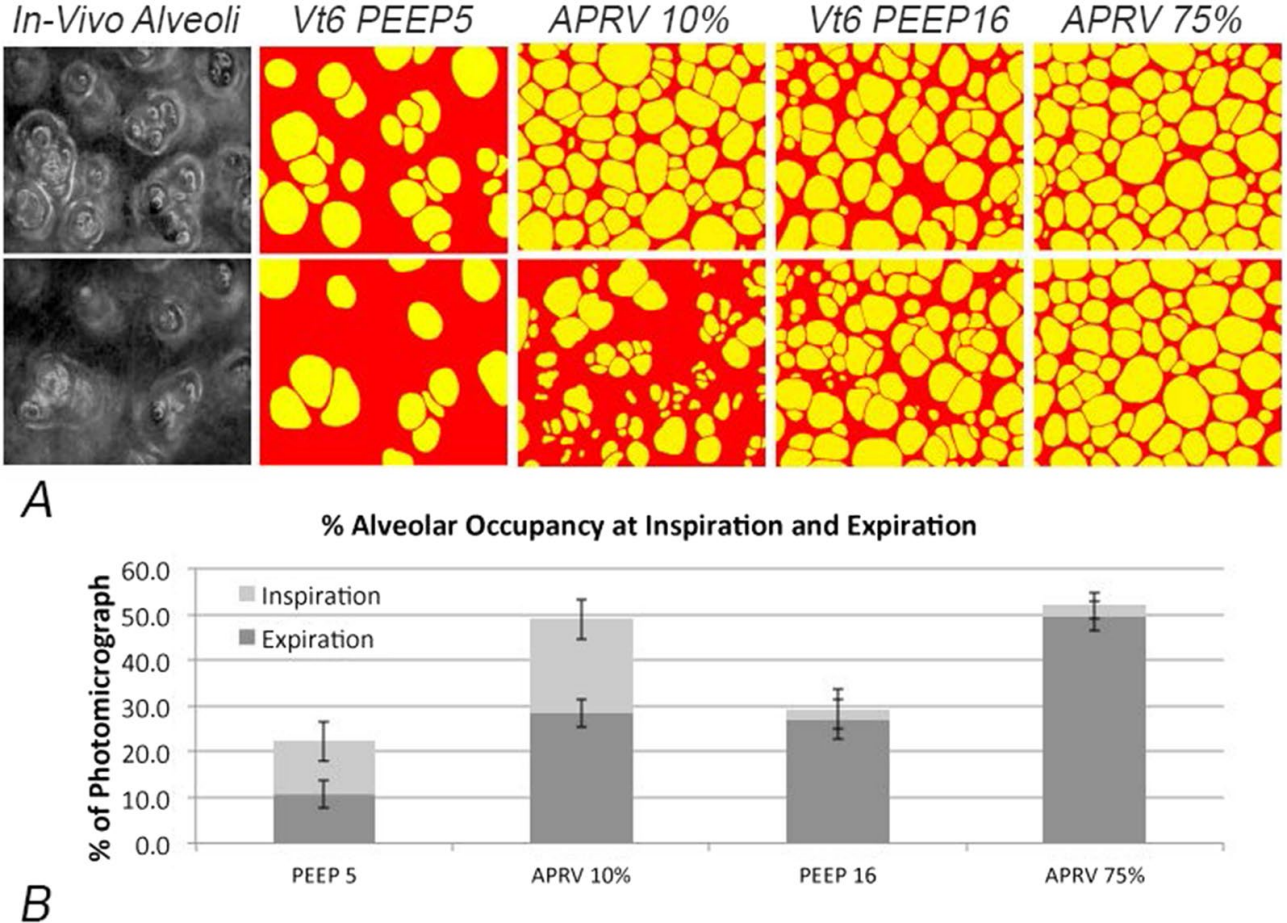
**A** Photomicrograph of in vivo subpleural alveoli (in vivo alveoli) at inspiration (top panel) and expiration (bottom panel) following lung injury in rats. Alveoli in the four ventilation treatment groups are depicted in yellow, while collapsed alveoli are in red. The images show the impact of ventilation strategies on alveolar recruitment and collapse using a conventional ventilation strategy with a low tidal volume (LV_T_) of 6 mL kg^−1^ (Vt6) combined with either PEEP 5 cmH_2_O (Vt6 PEEP5) or 16 cmH_2_O (Vt6 PEEP 16). Also shown for comparison are the results of using APRV with an extended *P*_High_ (CPAP Phase, Fig. 4B) combined with two expiratory durations set at either 75% of peak expiratory flow (APRV 75%) or 10% of peak expiratory flow (APRV 10%). APRV 75% has a very short expiratory duration, while APRV 10% has a much longer expiratory duration (Fig. 4B, Release Phase). **B** The impact of each ventilation strategy on alveolar recruitment at inspiration (light gray bar) and derecruitment at expiration (dark gray bar) expressed as the percent (%) of the microscopic field. Conventional ventilation using LV_T_ did not effectively recruit alveoli (PEEP5 and PEEP16). Increasing to PEEP16 reduced the number of alveoli that collapsed during expiration (difference between the light and dark gray bars), but did not recruit as many alveoli as APRV with an extended CPAP Phase. However, using APRV with an extended expiratory duration (APRV 10%) caused many of the newly recruited alveoli to re-collapsed. Alveolar collapse at expiration was prevented with the use of a brief expiratory duration (APRV 75%) [58]. (Permission to republish requested)

A mode of MV that potentially meets the above two requirements is APRV, although it must be administered in a very particular manner (Fig. 4B). The most critical APRV parameter is the duration of the expiratory Release Phase (*T*_Low_), which must be brief enough to prevent collapse of even the most rapidly closing alveoli, thereby attending to the first requirement. It is important to realize, however, that extending *T*_Low_ from its optimal value by even a fraction of a second can increase expiratory derecruitment dramatically, with potentially disastrous results for the lung (Fig. 6A, B—APRV 10%) [63].

The second requirement can be met by an appropriate level of inspiratory airway pressure (*P*_High_) applied for an extended duration (*T*_High_) (Fig. 4B, CPAP Phase). This effectively applies CPAP to the lung, initiating a gradual and sustained alveolar reopening over a prolonged period of time (Fig. 5B–D). Since the time required to recruit some regions of the lung can be extremely long, the benefits of opening the lung in this manner may not be evident for hours or even days (Fig. 5B–D) [32–35]. The level of pressure applied during expiration (*P*_Low_) is less critical to the derecruitment process because the brief *T*_Low_ prevents the lungs from completely emptying by the end of expiration, resulting in a degree of time-controlled PEEP (Fig. 4B, TC-PEEP). Setting *P*_Low_ to 0 cmH_2_O maximizes expiratory flow and facilitates CO_2_ elimination with each breath, thus helping to maintain normocarbia. At the exhalation termination point (*T*_Low_), the lung is rapidly re-inflated to the CPAP Phase (Fig. 4B, Gas Flow/Time curve, red arrowhead).

**Fig. 7:**
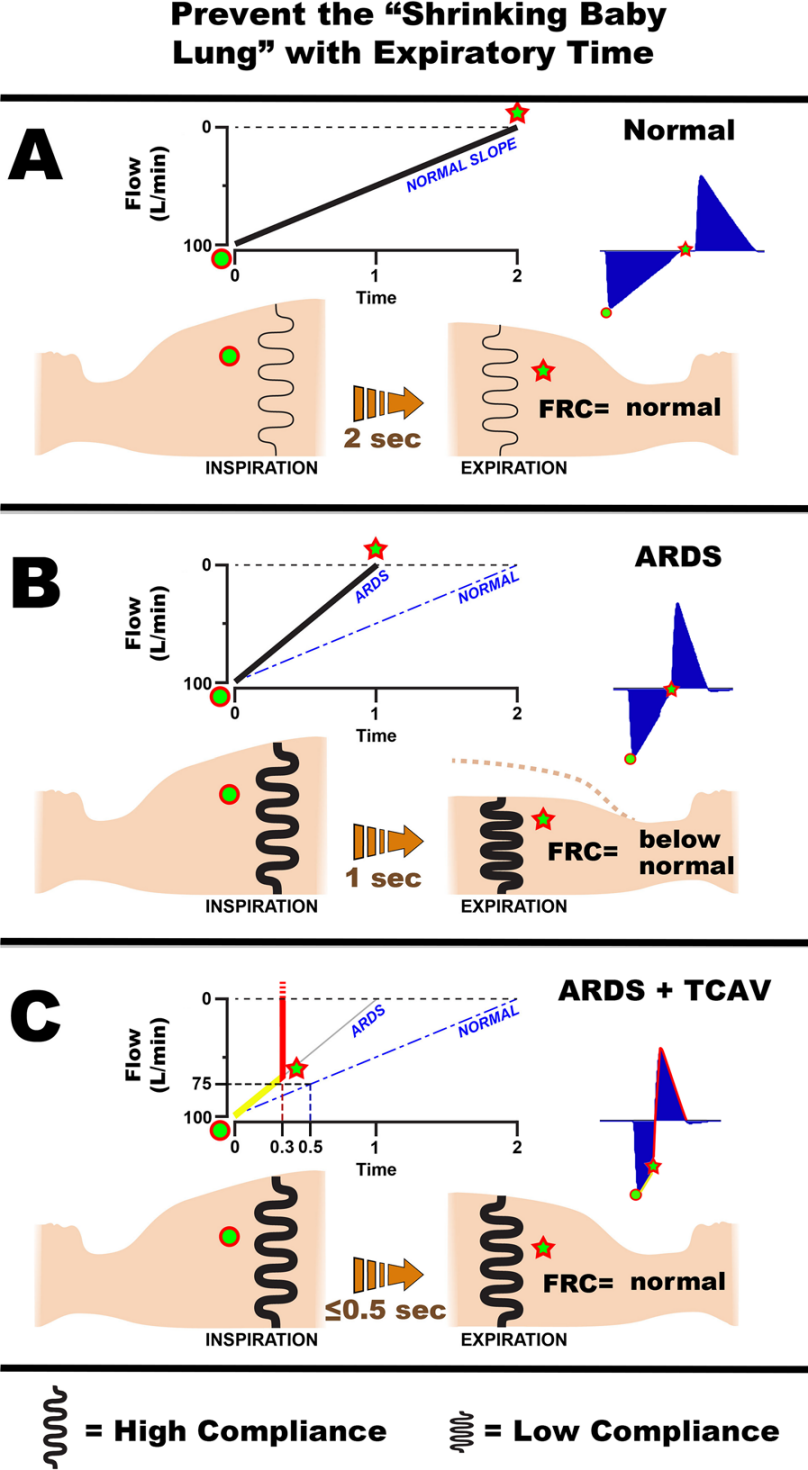
Using expiratory time and the airway pressure release ventilation (APRV) mode to maintain a normal functional residual capacity (FRC) and prevent progressive collapse of the ARDS lung (VILI vortex). **A** The Flow/Time curve (Fig. 4B) at the beginning of expiration (green dot) and at end expiration (Release Phase, red star). The normal lung is allowed to fully empty (Flow 0 L/min) to atmospheric pressure (0 cmH_2_O). The respiratory system compliance (*C*_RS_) in the normal lung is high, and therefore, the lung recoil is low (thin black spring). The slope of the expiratory flow curve (Slope_EF_) is shallow (NORMAL SLOPE) taking ~ 2 s for the lung to fully empty. A functioning pulmonary surfactant system prevents lung collapse at atmospheric pressure, and FRC remains normal. The Flow/Time curve that would be seen on the ventilator monitor in blue (Fig. 4B, Gas Flow/Time). **B** ARDS diminishes surfactant function and dramatically decreases C_RS_, leading to increased lung recoil (thick black spring). This results in a steep Slope_EF_ (ARDS, solid black line) compared with a normal slope (NORMAL, dashed blue line). As a result, the lung empties rapidly (~ 1 s), and there is a marked reduction in FRC seem as large reduction in chest volume (green star). The Flow/Time curve that would be seen on the ventilator monitor in blue (Fig. 4B, Gas Flow/Time). **C** The TCAV method uses a fraction (75%) of the peak expiratory flow (100 L/min) to set the expiratory duration. Changes in the Slope_EF_ with decreasing *C*_RS_ will modify the expiratory duration using this method (ARDS = 0.3 s; Normal = 0.5 s at the same 75% fraction). Note that in the ARDS lung the Slope_EF_ (yellow line) is steep and 75% is reached very rapidly (0.3 s) at which point the lung is rapidly re-inflated to the CPAP Phase (red line). In the normal lung, the Slope_EF_ is shallower and takes 0.5 s to reach 75% of the peak expiratory flow. The brief expiratory duration does not give the lung time to depressurize (Fig. 4B, TC-PEEP) or alveoli time to collapse (Fig. 6A, B, APRV 75%) maintaining a near normal FRC and preventing progressive lung collapse (VILI vortex). The Flow/Time curve that would be seen on the ventilator monitor in blue (Fig. 4B, Gas Flow/Time)

The values of *P*_High_, *T*_High_, *P*_Low_, and *T*_Low_ thus collectively define how APRV is administered. Using appropriate values for these parameters will mitigate some of the adverse effects of ventilating a baby lung. The question remains, however, as to how these values should be chosen, particularly *T*_Low_. Figure 7 shows: (A) normal expiratory flow/time curve, (B) the changes caused by ARDS, and (C) how this information can be used to set *T*_Low_ necessary to stabilize alveoli and maintain a normal functional residual capacity (FRC). Passive exhalation in the normal lung is slow (~ 2 s) due a high *C*_RS_ and low lung recoil (Fig. 7A—thin springs). A functioning pulmonary surfactant system maintains a normal FRC at atmospheric pressure. ARDS-induced surfactant deactivation reduces lung *C*_RS_ and increases recoil (thick springs) causing a rapid exhalation (~ 1 s) of gas during expiration (Fig. 7B—ARDS). The FRC can decrease by up to 45% in lungs with severe ARDS [64]. Thus, allowing the injured lung to collapse to atmospheric pressure will result in a much lower FRC than in the normal lung. Experience in both the animal laboratory and the intensive care unit (ICU) has demonstrated that the expiratory gas flow curve using the APRV mode can be used in a patient-targeted and adaptable approach to predict the *T*_Low_ duration necessary to stabilize alveoli.

Using this approach, *T*_Low_ is set such that expiration terminates when the magnitude of expiratory flow has fallen to a fixed percentage (75%) of its peak value at the start of expiration (Fig. 5C) [65, 66]. This results in *T*_Low_ values that vary across patients and disease states. For example, patients with severe ARDS typically have less compliant lungs that empty quickly, thus reaching their expiratory termination points (Fig. 7C, 0.3 s) earlier than patients with more compliant lungs (Fig. 7C, 0.5 s). This effect can be appreciated by examining the slope of the expiratory gas flow/time curve (Slope_EF_) from lungs with differing *C*_RS_ (Fig. 7C—ARDS and NORMAL) [67]. As lung health improves and *C*_RS_ increases, *T*_Low_ may be increased accordingly (Fig. 5C—ARDS = 0.3 s; NORMAL = 0.5 s). Thus, setting *T*_Low_ according to the Slope_EF_ affords a personalized approach to ventilating the injured lung that adapts to a patient’s changing physiology. Finally, similar to normalizing *V*_T_ to *C*_RS_ (i.e., driving pressure) to reduce mortality (Fig. 1B), *T*_Low_ adjusted to 75% of peak expiratory flow produces *V*_T_s (Fig. 5B 6.2 mL/kg and Fig. 5C 9.2 mL/kg) that is proportional to *C*_RS_ (Fig. 5B = *C*_RS_ low; 5D = *C*_RS_ high).

Applying APRV in the above manner requires continuous monitoring of intra-breath expiratory flow as well as the ability to control the termination of expiration. This precise method of setting the APRV mode using the Slope_EF_ is termed the *time-controlled adaptive ventilation* (TCAV) method (Fig. 7C). The TCAV method has been shown to enhance lung protection [63, 66, 68–83] by stabilizing alveoli [63] (Fig. 6A, B—APRV 75%) and progressively reopening recalcitrant regions of atelectasis (Fig. 5 B–D). TCAV rapidly pulls the lung out of the VILI vortex and then gradually reopens collapsed tissue (Fig. 2) [63, 70, 84].

The TCAV method has been extensively studied by several groups [63, 66, 68, 71–83, 85, 86]. It has been shown to recruit subpleural alveoli in a rat model of ARDS (Fig. 6, APRV 75%) [63, 71, 86] and reduce tissue damage compared to ARDSNet LV_T_ strategy in a clinically applicable porcine sepsis and gut ischemia/reperfusion-induced ARDS model [72, 79]. The TCAV method has also been shown to reduce ARDS incidence and mortality in trauma patients [60]. These findings suggest the physiologic principles upon which TCAV is based represent a successful method to balancing the benefits of positive pressure ventilation against the harm it may cause to an already injured lung.

It must be noted that in patients with expiratory flow limitations (EFL) the TCAV method must be modified. Since flow/time is an integral of volume, airflow limitations are easily depicted with changes in peak expiratory flow rates and can be readily seen at the bedside with flow graphics using the TCAV method. The pattern of airflow limitations results in the following characteristics: (a) The peak expiratory flow rate decreases as expected in diseases with obstructed airways and (b) an uneven pattern of incomplete and sequential gas emptying that greatly increases the deceleration angle of the expiratory flow curve, which is a hallmark of obstructive lung disease. Once EFL is identified, the TCAV protocol is modified to increase the expiratory duration, since more time is needed for the same volume of gas to be exhaled. The TCAV protocol for patients with EFL is beyond the scope of this paper.

**Fig. 8:**
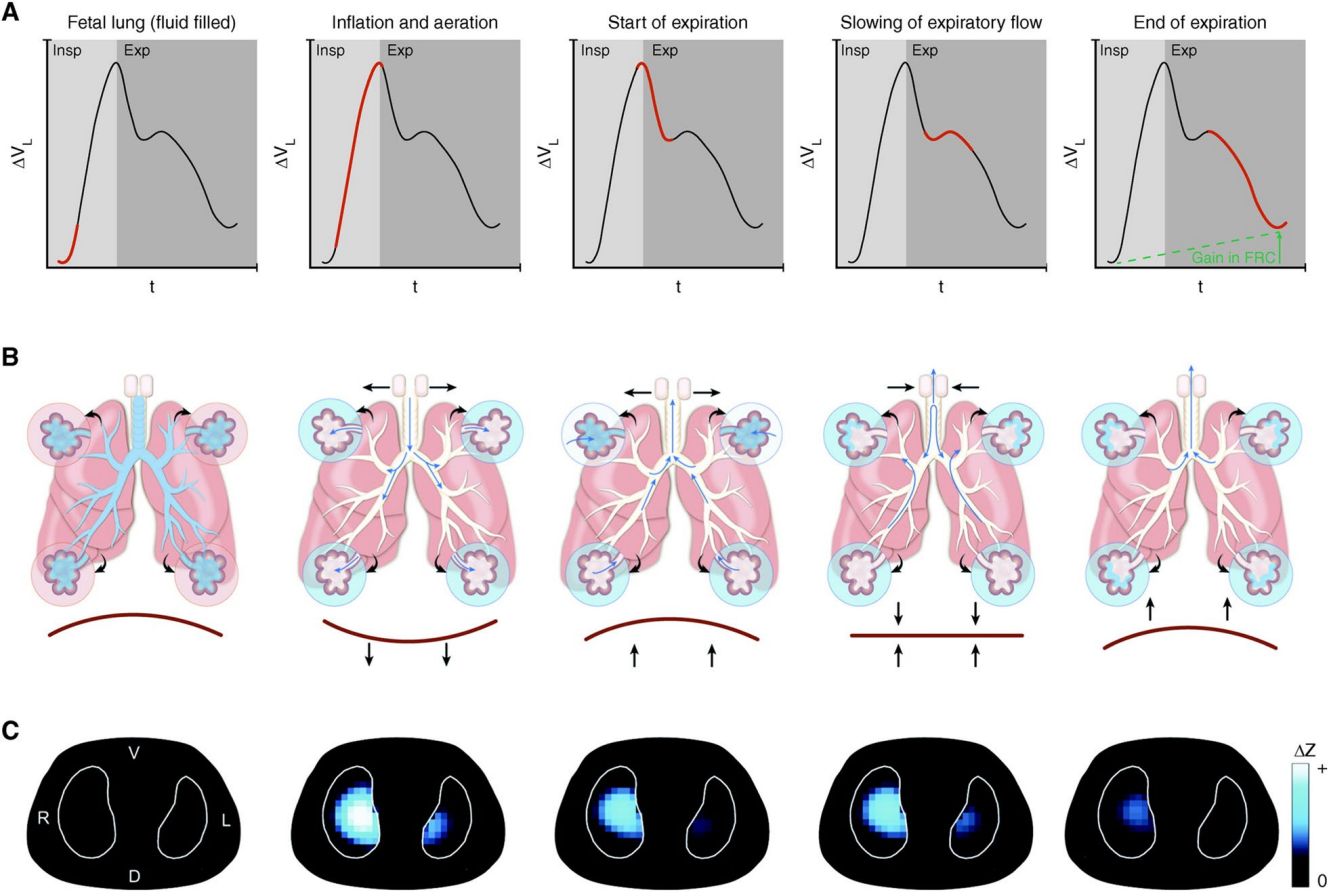
Five phases of a newborn infant transitioning from a collapsed fluid-filled state to full aeration. **A** Lung volume/time curve representing a single ‘cry’ during inspiration (Insp) and expiration (Exp). **B** Diagram of airways and acini. **C** Electrical impedance tomography (EIT) images. (1) *Fetal lung (fluid filled)*: The beginning of the newborn’s first inspiration (red line) with the lung still collapsed and fluid filled (blue in airways) and no gas in the lung measured with EIT (no blue). (2) *Inflation and aeration*: A rapid inspiration (red line) with the glottis fully open (horizontal arrows pointing out) and contraction of the diaphragm (downward arrows) as the newborn rapidly fills the lung with gas to begin a cry. This high velocity inflow of air moves liquid in the airways and alveoli (blue changing to white) into the interstitial space (small blue arrows). EIT shows gas entering the lung (light blue). (3) *Start of expiration*: Expiration is active with diaphragm contraction (arrows) forcing gas rapidly out of the lungs (red steep slope, volume/time curve). Intra-alveolar pressure falls, allowing fluid to refill into some alveoli (upper alveoli going from white to blue) and EIT shows a loss of lung volume. (4) *Slowing of expiratory flow*: To prevent further lung collapse and flooding, the glottis briefly ‘brakes’ expiratory gas flow (horizontal arrows pointing inward), re-pressurizing the lungs. Pendelluft (blue arrows) redistributes gas into partially flooded alveoli. (5) *End of expiration*: The remainder of expiration (red line on volume/time curve) occurs with a partially closed glottis to maintain a PEEP to preserve FRC. EIT demonstrates that FRC is preserved (blue areas). Thus, the newborn uses a rapid inspiration to open flooded tissue and partially closes the glottis as a brake to prevent re-collapse and flooding. This method of rapid inspiration to open lung and closing the glottis to ‘brake’ expiratory flow and maintain airway pressure to prevent re-collapse *ratchets* open small volumes of lung tissue with each breath until the lung is fully inflated. The TCAV method uses a similar *ratchet approach* to open collapsed and fluid-filled lung of the ARDS patient. When expiratory flow is terminated (Fig. 4B, Gas Flow/Time curve, red arrowhead), the lung is rapidly re-inflated until the set CPAP Phase pressure is reached. This is analogous to the rapid inspiration before a cry in the newborn (*Inflation and aeration)*. The brief Release Phase (0.2–0.5 s) acts as an expiratory ‘brake’ (Fig. 4B, red arrowhead), which does not allow the lung to depressurize, maintaining a time-controlled PEEP (Fig. 4B, TC-PEEP). This is similar to the newborn using the glottis as a ‘brake’ to slow expiratory flow (*Slowing of expiratory flow)*. To summarize, both the newborn and the TCAV method use a rapid inspiration to open a small volume of lung with each breath. A ‘brake’ in expiratory flow is used to prevent re-collapse. Combined this ratchet approach opens small volumes with each breath and over time fully recruits the collapsed lung

Insight into the mechanism by which the TCAV method opens collapsed lung tissue can be gained by analyzing the method the newborn infant uses to open their collapsed lung at birth (Fig. 8) [87]. Spontaneously breathing newborns recruit collapsed lung with a rapid inspiration (Fig. 8—*Inflation and aeration*) and prevent re-collapse of the newly opened tissue by partially closing the glottis to act as a ‘brake’ on expiratory flow (Fig. 8—*Slowing of expiratory flow*). Thus, the method by which nature opens the lung at birth is to inflate a small amount of tissue with each breath and then apply a ‘brake’ to prevent the newly opened tissue from re-collapsing. When a force causes a strain in one direction and some type of ‘brake’ prevents strain in the opposite direction it is termed a *ratchet*. Therefore, nature’s strategy is to ratchet open lung tissue with each breath until the lung is fully inflated.

We postulate that the TCAV method uses the ratchet mechanism to open collapsed lung in the ARDS patient. There is a rapid lung inflation from the termination point of expiration (Fig. 4B, Gas Flow/Time curve) that recruits a small portion of collapsed lung, combined with a brief Release Phase to ‘brake’ expiratory flow preventing derecruitment during expiration. In addition, the prolonged CPAP Phase accelerates recruitment of alveoli with long opening time constants (Fig. 4B, CPAP Phase). Addition of CPAP has been shown to significantly increase FRC in premature infants [88].

If our hypothesis is correct, the *ratchet approach* is a novel and innovative method of lung recruitment. Unlike the OLA, which attempts to open the entire lung in a seconds or minutes [89], the TCAV method ratchets open small volumes of lung with each breath that will progressively open the entire lung over hours or days. As described above a ratchet is a device (ventilator) that causes an object to strain in one direction (alveolar recruitment during inspiration), while applying a ‘brake’ (very short expiratory duration) necessary to prevent strain in the opposite direction (prevent alveolar collapse during exhalation). An example of the ratcheting approach on progressive lung recruitment is shown in Fig. 5.

## Conclusions

Low tidal volumes and airway pressures using the ARDSNet method can push the lung with mild ARDS into the VILI vortex. To prevent progressive lung collapse, the time-dependent nature of alveolar opening and collapse must be taken into account. The TCAV method to set APRV uses: (i) the ratchet approach combined with an extended inspiratory duration necessary to recruit alveoli and (ii) a brief expiratory duration to ‘brake’ the derecruitment of rapidly collapsing alveoli. The TCAV method is personalized and adaptable and has shown promising results in animal models of ARDS and has yielded positive clinical outcomes in the ICU. Whether TCAV, or a similar strategy, can significantly reduce ARDS-related mortality when implemented in a large-scale clinical trial remains to be seen, but it is clear that a new approach to MV in ARDS is needed. We propose that ventilating patients with ARDS in a manner that specifically addresses the time dependence of R/D is a logical strategy for interrupting and reversing the VILI vortex. Accordingly, we believe consideration should be given to the design of a clinical trial comparing the TCAV method to the current standard of care in ARDS patients.

## Data Availability

Not applicable.
